# Calculation of Strain Energy Density Function Using Ogden Model and Mooney–Rivlin Model Based on Biaxial Elongation Experiments of Silicone Rubber

**DOI:** 10.3390/polym15102266

**Published:** 2023-05-11

**Authors:** Yoshihiro Yamashita, Hideyuki Uematsu, Shuichi Tanoue

**Affiliations:** 1 Research Center for Fibers and Materials, University of Fukui, Fukui 9110-8507, Japan; 2 Frontier Fiber Technology and Science Faculty of Engineering, University of Fukui, Fukui 9910-8507, Japan

**Keywords:** nonlinear, hyperelastic, strain energy density function, FEM, biaxial deformation

## Abstract

Strain energy density functions are used in CAE analysis of hyperelastic materials such as rubber and elastomers. This function can originally be obtained only by experiments using biaxial deformation, but the difficulty of such experiments has made it almost impossible to put the function to practical use. Furthermore, it has been unclear how to introduce the strain energy density function necessary for CAE analysis from the results of biaxial deformation experiments on rubber. In this study, parameters of the Ogden and Mooney–Rivlin approximations of the strain energy density function were derived from the results of biaxial deformation experiments on silicone rubber, and their validity was verified. These results showed that it is best to determine the coefficients of the approximate equations for the strain energy density function after 10 cycles of repeated elongation of rubber in an equal biaxial deformation state, followed by equal biaxial elongation, uniaxial constrained biaxial elongation, and uniaxial elongation to obtain these three stress–strain curves.

## 1. Introduction

CAE analysis has become indispensable for the development and design of new products [1,2,3,4,5,6,7,8,9,10,11,12,13,14,15,16]. Abaqus and Marc are well-known analytical software programs, but recently Ansys and Nastran have also become popular. LS-DYNA is often used for crash analysis. Materials such as rubbers and elastomers with strains of more than 100% are called hyperelastic materials. In CAE analysis of such materials, it is difficult to simulate their behavior using linear analysis based on Young’s modulus and Poisson’s ratio, which are used for metals, glass, and plastics, so it is common to define a hyperelastic material by its strain energy density function. The Mooney–Rivlin model [17,18,19,20,21] and the Ogden model [22] are well-known approximations to express this function. We have proposed a Mooney–Rivlin model that reflects the internal structures of rubber materials [23].

Kawabata [19,20,21], Yamashita [23], Urayama [24,25], and Fujikawa [26] have been working intensively on biaxial testing. Regarding the relationship between biaxial deformation and rubber elasticity, Shen et al. developed a unified framework to describe the superelasticity and damage behaviors of rubber materials [27]. Akbari et al. proposed a model to describe the strain energy functions of rubber materials based on variables corresponding to polymer chain dynamics [28]. S. K. Melly et al. reported that the Yeoh model underestimates stresses during equal biaxial loading [29]. Khiem et al. provided a complete thermomechanical characterization of crystallized filled natural rubber under uniaxial and biaxial loading [30].

In addition to models to predict rubber material behavior under uniaxial and biaxial loading, Arunachala et al. validated models to quantitatively estimate the effect of crystallization on fracture initiation [31]. Liao et al. showed that in uniaxial, pure shear, and equal biaxial tensile tests of silicone rubber, as well as in Shore hardness with different strain levels, stress softening can be significantly recovered with time [32]. Ansari-Benam et al. proposed a new model of a generalized Hookean strain energy function for application to finite deformation of incompressible elastomers based on biaxial test results [33]. VanArsdale developed the constitutive equation for rubber-like materials using the tensor of elongation based on biaxial elongation experimental results [34]. Rajesh et al. used the Mooney–Rivlin material model to estimate material coefficients using biaxial test results and studied the structural integrity of propellant particles under various loading conditions using ANSYS numerical simulation software [35]. Blaise et al. also constructed a new model to validate the nonlinear elastic response of rubber-like materials [36]. Pourmodheji et al. applied the same energy-based argument to thin film fracture, focusing on the plural growth modes, the first one that gives the maximum critical load based on biaxial tests of natural rubber and styrene-butadiene rubber [37]. Chen et al. reported strain-induced crystallization of natural rubber by biaxial stretching [38]. Lopez-Campos et al. represented a viscoelastic model of rubber-like materials using a classical hyperelastic model (Mooney–Rivlin) coupled with a nonlinear viscous part involving both strain and time dependence [39]. Sadeg et al. reported that the failure of polymers at the macroscopic level under biaxial elongation is a consequence of the presence of internal defects [40]. Urayama et al. investigated the Mullins effect in filled elastomers by biaxial deformation in detail and considered that this effect reflects the fracture process of the intrinsic structure, including the filler network and the filler–polymer interface as well as the friction between the filler and rubber matrix [24]. Using coarse-grained molecular dynamics simulations, Uddin et al. studied a multiscale scheme for constitutive modeling of natural rubber by fitting biaxial test results [41]. Iba et al. studied the nonlinear stress–strain behaviors of highly porous elastomeric foams by several forms of biaxial and uniaxial tensile and compression [25]. Anssari-Benam et al. discussed the central role of invariant *I*_2_ in biaxial deformation in nonlinear elasticity [42]. Zheng et al. used biaxial tests to systematically compare the effects of chain entanglement on the elasticity and viscoelasticity of polymer networks with the relationship between a polymer network failure and fatigue [43].

These biaxial test measurements of rubber are used to validate the entanglement model of rubber molecular chains and their viscoelastic properties, but few studies have related them to the strain energy density function for CAE analysis. Biaxial measurements are essential for obtaining mechanical data of rubber needed for CAE analysis. Only a few companies worldwide offer such measurement instruments on the market. These include the EX10-B biaxial stretching tester from Toyo Seiki, the BX5450 biaxial tensile tester from Katotech, and the 11A9 film biaxial stretching machine from Imoto Manufacturing Co. Among these, only the BX5450 is suitable for measuring the superelasticity of rubber for CAE analysis.

The advantage of this device is that it can obtain stress–strain curves of a 0.5 mm thick, 70 mm square rubber sheet under uniform biaxial deformation up to 300%, and it can change the tensile speed from 0.01 mm/s to 10 mm/s. Additionally, it can operate at temperatures ranging from −30 °C to 80 °C by attaching a thermostatic chamber.

In this study, the Ogden and Mooney–Rivlin coefficients, which are commonly used in CAE analysis, were calculated by cyclic elongation of silicone rubber using a biaxial tensile tester from Kato Tec Corporation, which is used as a worldwide standard, and their practicality was verified. Moreover, even with the introduction of biaxial testing equipment, there is little knowledge of how the data for CAE analysis can be compared with actual measurement results of the product under repeated tensile data measurement conditions. We also examined how the biaxial test results can be used for CAE analysis.

## 2. Materials and Methods

### 2.1. Silicon Rubber

The silicone rubber used for this experiment was KE-1950-30-A/B (hardness 31) made by Kyowa Kogyou Co. LTD (Saitama, Japan). The curing conditions of the addition curing type were 120 °C for 5 min, followed by molding at 150 °C for 1 h using a mold (length and width 10.0 mm in length and width, 0.5 mm in thickness) [44].

### 2.2. Biaxial Tensile Tester

**Figure 1:**
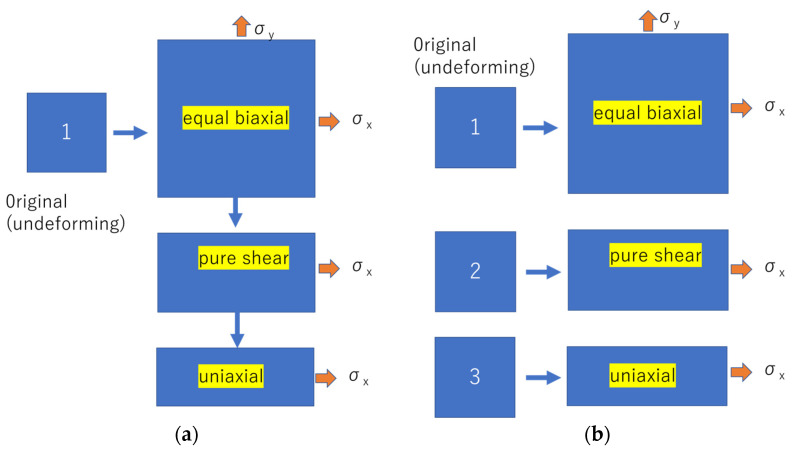
Biaxial deformation test method. (**a**) Three deformations are performed sequentially on the same sheet; (**b**) three deformations are performed on separate sample sheets.

BX5450 biaxial elongation tester manufactured by Kato Tech was used [45]. The sample size was 70 mm × 70 mm with a thickness of 0.50 mm. The distance between chucks was 50 mm, and the tensile speed was 2 mm/s (strain rate 2.4/min). The measurement atmosphere was room temperature (25 °C) and humidity 50%. The experimental procedure is shown in Figure 1.

### 2.3. Strain Energy Density Function (W) [46]

Rubber elasticity can be considered phenomenologically in terms of the theory of elastic deformation of continua. For a homogeneous isotropic elastic body, *W* is a symmetric function of the principal axis elongation ratio *λ_i_*_,_ (*i* = 1, 2, 3) and can also be expressed as an invariant of the deformation tensor *I_i_* (*i* = 1, 2, 3). That is,

(1) $$W=W\left(\lambda_{1},\lambda_{2},\lambda_{3}\right)$$

(2) $$W=W\left(I_{1},I_{2},I_{3}\right)$$

*I_i_* is expressed using *λ_i_* as follows.

(3) $$I_{1}=\lambda_{1}^{2}+\lambda_{2}^{2}+\lambda_{3}^{2}$$

(4) $$I_{2}=\lambda_{1}^{2}\lambda_{2}^{2}+\lambda_{2}^{2}\lambda_{3}^{2}+\lambda_{3}^{2}\lambda_{1}^{2}$$

(5) $$I_{3}=\lambda_{1}^{2}\lambda_{2}^{2}\lambda_{3}^{2}$$

Since most rubber crosslinkers are nearly incompressible to deformation, *W* can be expressed as a function of *I*_1_ and *I*_2_ if *I*_3_ = 1. Mooney–Rivlin used this to define an approximate expression for the strain energy density function when the volume is unchanged by deformation in Equation (6).

(6) $$W=\overset{N}{\underset{p,q=0}{\sum }}C_{pq}{\left(I_{1}-3\right)}^{p}{\left(I_{2}-3\right)}^{q}$$

In this study, we used the James–Green–Simpson [47] Equation (7) and the Yamashita [22] Equation (8) in this expansion formula. Ogden [23] also proposed Equation (9) as an approximate formula. In this study, this equation was expanded up to *N* = 3 and used.

(7) $$W={\;C}_{10}\left(I_{1}-3\right)+C_{01}\left(I_{2}-3\right)+{\;C}_{11}\left(I_{1}-3\right)\left(I_{2}-3\right)+C_{20}{\left(I_{1}-3\right)}^{2}+C_{30}{\left(I_{1}-3\right)}^{3}$$

(8) $$W=C_{10}\left(I_{1}-3\right)+C_{01}\left(I_{2}-3\right)+C_{nm}\frac{{\left(I_{1}-3\right)}^{n}}{{\left(I_{2}-3\right)}^{m}}$$

(9) $$W=\overset{N}{\underset{p=1}{\sum }}\frac{\mu^{p}}{\alpha^{p}}\left(\lambda_{1}^{\alpha^{p}}+\lambda_{2}^{\alpha^{p}}+\lambda_{3}^{\alpha^{p}}-3\right)$$

In Japan, the largest number of researchers and engineers use the Ogden Equation (9), followed by those who use the Mooney–Rivlin Equation (6). Some of the model equations that have been improved on these models are Equations (7) and (8). Principal stress σ_x_ can be determined by differentiating strain energy by strain. In this section, only the stress in the extensional direction is treated.

### 2.4. CAE Simulation

Model calculations were performed using Abaqus/CAE6.14, the world’s most commonly used general purpose software capable of performing hyperelastic finite element analyses using the Ogden and Mooney–Rivlin coefficients.

## 3. Results

### 3.1. Comparison of Stress (σ_x_)–Strain (ε Curves for Equal Biaxial, Uniaxially Constrained Biaxial, and Uniaxial Deformation on the Same Sample and for Different Samples

**Figure 2:**
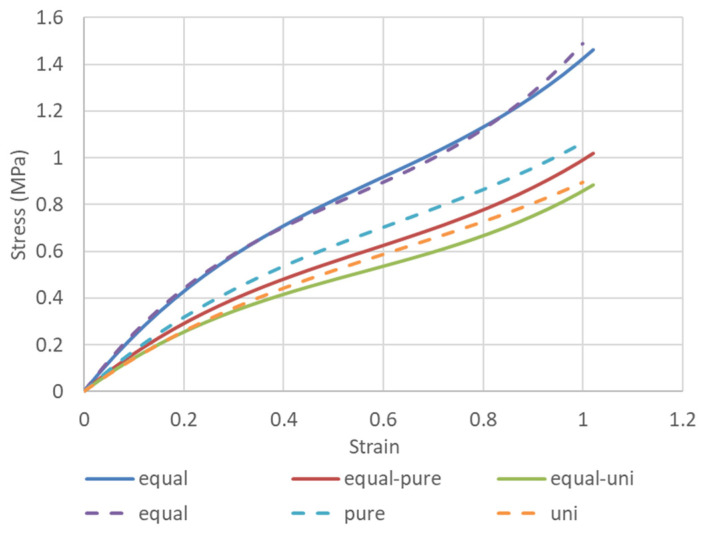
Stress–strain curves for the same sample subjected to different biaxial deformations (dashed lines) vs. those for separate samples subjected to biaxial deformations (solid lines).

The solid line in Figure 2 shows a case in which the same sample was subjected to equal biaxial deformation to 100% strain, followed by uniaxially constrained biaxial deformation and finally by uniaxial deformation (Figure 1a model). The dashed lines in Figure 2 show the case where three different samples were individually subjected to equal biaxial deformation, uniaxially constrained biaxial deformation, and uniaxial deformation (Figure 1b model). The results show that when the same sample is used, the first equal biaxial deformation causes a strong unraveling of polymer chain entanglement and the rupture of crosslinking points, whereas the uniaxially constrained biaxial and uniaxial deformations are performed while maintaining these conditions. In contrast, it is easy to assume that if separate samples are used for equal biaxial elongation only, uniaxially constrained biaxial elongation only, or uniaxial elongation only, the polymer chains on the side not undergoing deformation will not unravel, and the stress values will be greater than the results for the same sample with the unraveling of the polymer chains of the X and Y directions. The Ogden and Mooney–Rivlin coefficients were obtained from both results and are compared in Table 1.

The Ogden coefficients in Table 1 are characterized by the fact that *μ*_1_ and *μ*_2_, which give the overall stiffness, are smaller than those obtained by elongation in only one direction because the entanglement of the molecular chains is greatly loosened by first performing uniform equal biaxial deformation. Similarly, only C_10_ appears to be involved in the Mooney–Rivlin coefficient. This means that estimating the Ogden and Mooney–Rivlin coefficients from uniaxial elongation data alone would lead to a stiffer estimation of the material than the actual product, as Anssari-Benam Afshin et al. [42] have stated that entanglement is related to the *I*_2_ term, whereas in the present study, the converse is true: C_01_ and C_11_ are related to the *I*_2_ term. The *I*_2_ term is slightly larger for the polymer chains that have been unraveled by equal biaxial elongation. Kawabata et al. [19,20,21,22] also stated that the *I*_2_ term is related to the entanglement of polymer chains, so further verification is needed.

### 3.2. Change in Ogden and Mooney–Rivlin Coefficients Due to Repeated Deformation in Equal Biaxial, Uniaxial Constrained Biaxial, and Uniaxial Elongation

**Figure 3:**
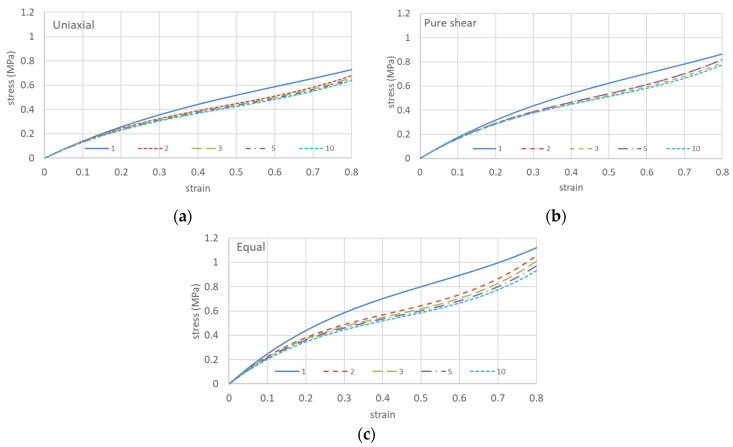
Ten cycles of repeated elongation in the same direction only. (**a**) Uniaxial; (**b**) pure shear; (**c**) equal biaxial.

Figure 3 shows the results of 10 cycles of repeated elongation in the same direction only.

As shown in Table 2, respectively, the Ogden coefficient and the Mooney–Rivlin coefficient were calculated from the stress–strain curves of equal biaxial, uniaxially constrained biaxial, and uniaxially elongated specimens for the first, second, third, fifth, and tenth repetitions. Evaluation of the results for these two coefficients shows that the Ogden coefficients change randomly while the Mooney–Rivlin coefficients C_10_ and C_11_ decrease with repetitive elongation. The other coefficients show little change. This is consistent with the fact that the entanglement unraveling is reflected in C_10_ and C_11_, but not in C_01_ related to the *I*_2_ term, as in the biaxial elongation for the same and different samples in Table 1b.

**Figure 4:**
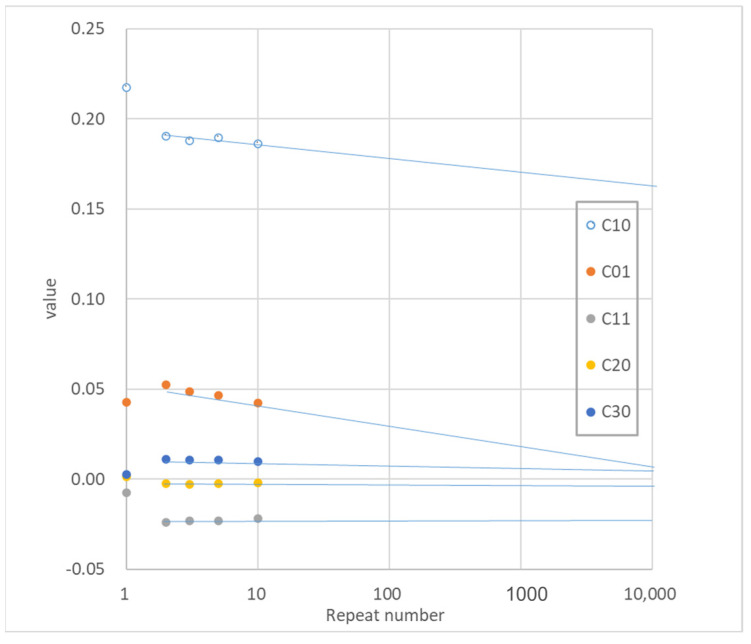
Assuming the decay of Mooney–Rivlin coefficients with the number of iterations.

**Figure 5:**
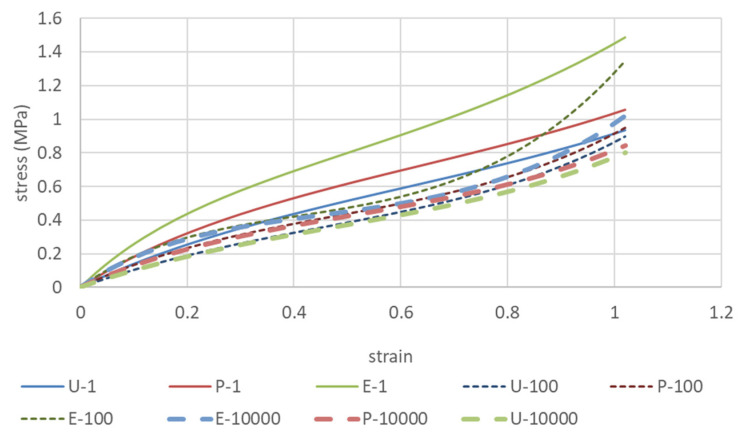
Predicted biaxial deformation stress–strain curves at repeated elongation obtained from extrapolation of the Ogden coefficient in Figure 4.

Therefore, assuming the decay of these coefficients with the number of iterations (Figure 4), the coefficients for the 100th and 10,000th iterations are shown in Table 3, and the predicted stress–strain curves are shown in Figure 5.

Since actual products are used repeatedly, it is practical to predict the actual deformation state of the product using the steady-state stress–strain curve. In reality, it takes about one week to do 10,000 repetition tests, so it is necessary to calculate the coefficients for the strain energy function at a steady state to some extent in this practical manner.

### 3.3. Coefficients When Sagging Is Removed after Repeated Elongation and Measurement Is Performed Again

**Figure 6:**
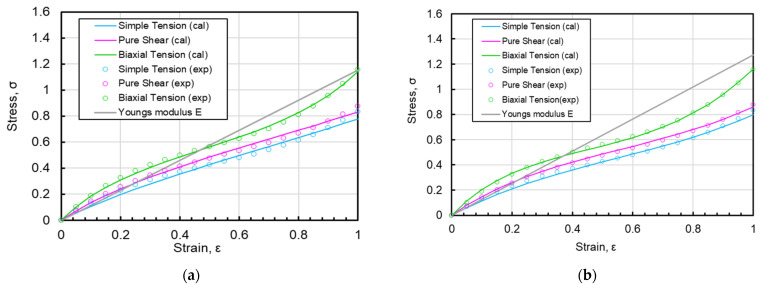
Equal biaxial, uniaxial constrained biaxial, uniaxial strain curve at 11th elongation after 10 repetitions under equal biaxial conditions, (**a**) approximation by Ogden function; (**b**) approximation by Mooney–Rivlin function.

Since it is difficult to repeat elongation 10,000 times in practice, we are now providing the coefficients of the strain energy density function for the sample measured at the 11th time after the sample has been elongated 10 times in succession, relaxed for 24 h, and then set again to remove the initial sag. The coefficients currently used are the 11th coefficients measured after the sample has been elongated 10 times by equal biaxial and after the initial sag due to relaxation has been removed. The agreement between the measured and approximated Ogden’s third order is shown in Figure 6a. Similarly, the agreement between the Mooney–Rivlin measurement and the approximation is shown in Figure 6b, which reveals that the accuracy of both approximations is almost the same. Table 4 shows the coefficients of the 11th iteration after repeating the equal biaxial 10 times and the coefficients of the 11th iteration after repeating the uniaxial elongation, uniaxial constrained biaxial elongation, and equal biaxial elongation individually 10 times, each in the respective deformation style.

**Figure 7:**
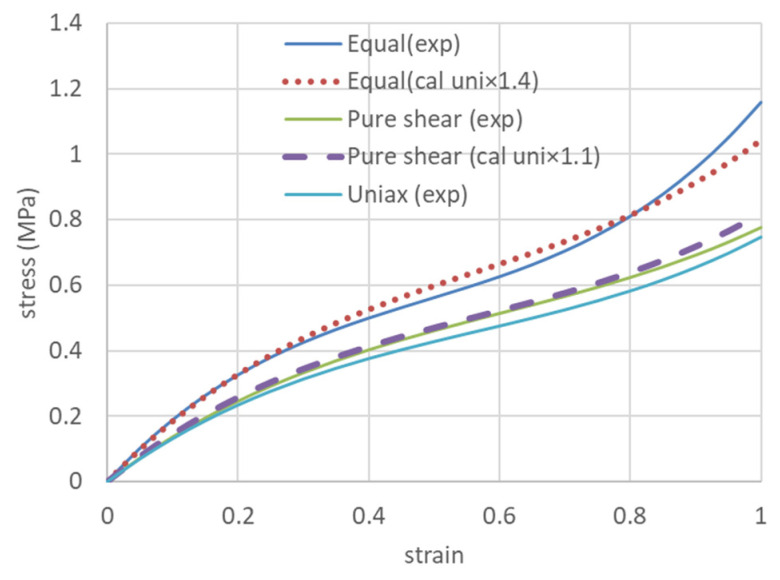
Stress values of experimental data for uniaxial elongation were multiplied by 1.1 and compared with experimental data for pure shear (uniaxially constrained biaxially), and by 1.4 and compared with experimental data for equally biaxial.

Yamashita et al. [48] also proposed a simple prediction based on the similarity of stress–strain curves when biaxial testing is difficult. That is, for the uniaxially constrained biaxial stress–strain curve, the uniaxial stress value is multiplied by 1.1, and for the equi-biaxial stress–strain curve, the uniaxial stress value is multiplied by 1.4. These values vary depending on the type of rubber, the crosslink density, and the amount of reinforcement, so these values are averages. In this study, this value was used to predict uniaxial stress–strain curves for uniaxially constrained biaxial and equidistant biaxial specimens, and very good agreement was obtained (Figure 7). The error was 3.6% for the uniaxially constrained biaxial stress–strain curve and 4.2% for the equally constrained biaxial stress–strain curve. This may be because the silicone rubber used is a rubber-like elastic material with a small amount of reinforcement.

### 3.4. Variation of the Strain Energy Density Function with Repeated Elongation

Although we have focused on the Ogden coefficient and the Mooney–Rivlin coefficient, these values are the only coefficients in the approximation equation to obtain the strain energy density function, and they provide optimal values only to improve the accuracy of the approximation. We found it unlikely that the Ogden coefficient reflects the internal structure of the rubber material. On the other hand, since the strain energy density function is the energy stored inside the rubber when it is deformed, this functional system should be discussed. However, little discussion has appeared in the literature about the strain energy function of rubber.

The relationship between Ogden’s coefficient and the strain energy density function is expressed by Equations (6)–(9). The advantage of the Ogden coefficient is that it defines the strain energy density function as a function of the elongation ratio, which has the advantage of being more intuitively understandable than *I*_1_ and *I*_2_, the universal quantities of Mooney–Rivlin’s elongation ratio.

**Figure 8:**
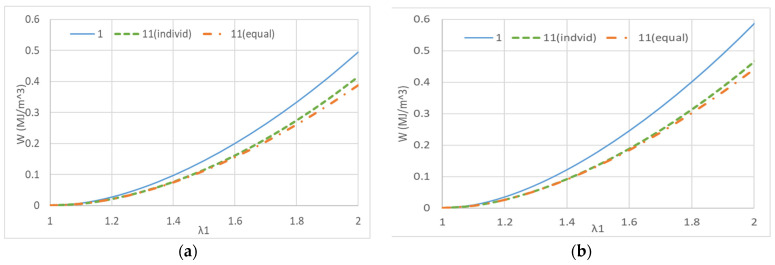

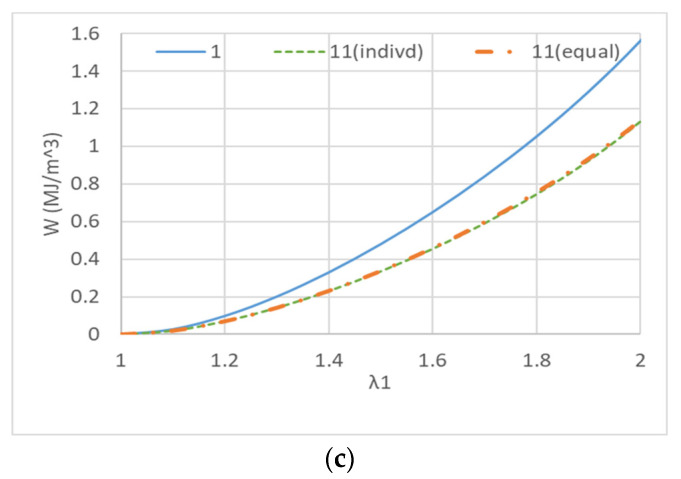
Change in strain energy density function values between unstretched and after 10 cycles of repeated elongation calculated based on Ogden coefficient: (**a**) Uniaxial deformation; (**b**) pure shear deformation; (**c**) equal biaxial deformation.

Therefore, the strain energy density functions obtained from Table 2a, Table 3a, and the 11th coefficient in the table are calculated for uniaxial elongation, uniaxial constrained biaxial elongation, and equal biaxial elongation, respectively. The results are shown in Figure 8.

The results show that repeated elongation causes the entanglement between molecules to unravel and the strain energy to gradually decrease. In addition, the uniaxial elongation is different from the uniaxial elongation after repeated equal biaxial elongation in terms of the entanglement unraveling. In other words, it makes sense that the entanglement in the unelongated direction does not unravel, and therefore the strain energy is higher than in the case of uniform repetitive elongation. To obtain the energy, the strain energy function from the equal biaxial elongation was integrated over the elongation ratio range of 1 to 2, and a comparison of the strain energy between the 1st and 11th cycles of equal biaxial elongation revealed a 24.5% decrease. In other words, 25% of the mechanical behavior of rubber involves the entanglement of rubber molecular chains and the breakdown of cohesion of reinforcing silica, other than rubber elasticity. Similarly, in the case of uniaxial elongation, the reduction is 18.5% for uniaxial elongation only but 21.9% for uniaxial elongation after repeated equal biaxial elongation. We estimate that 3.4% of this difference corresponds to entanglements that were not loosened by uniaxial elongation only. Similarly, the uniaxially restrained biaxial elongation resulted in a 23.6% decrease when compared to the uniaxially restrained biaxial elongation after a repeated 22.8% equal biaxial elongation, but the difference was 0.8%, which was smaller than that of uniaxial elongation. This indicates that even though the molecular chains are constrained, they are loaded with elongation and the entanglement on the constrained side is loosened.

**Figure 9:**
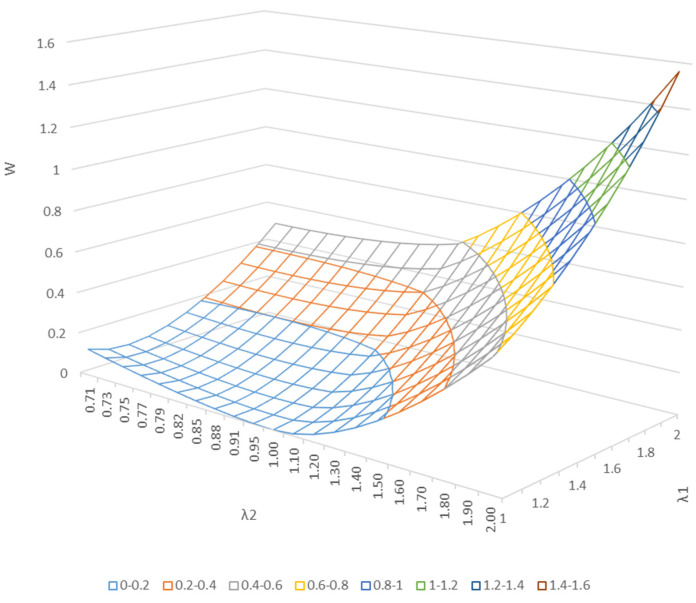
Three-dimensional plot of the relationship between the free energy per unit volume (strain energy density function (*W*)) stored in a material when the material is deformed by an arbitrary amount and the elongation ratio *λ* in biaxial deformation.

Figure 9 shows the strain energy density function after 11 cycles of stretching in the cyclic equidirectional biaxial direction, displayed as a three-dimensional surface. We believe that discussing the properties of rubber materials in terms of strain energy, rather than in terms of stress, is meaningful for understanding the relationship between biaxial deformation and the internal structure of rubber.

## 4. Discussion

### 4.1. Effect of Polymer Chain Entanglement on Strain Energy Density Function

The Ogden and Mooney–Rivlin coefficients have been determined under conditions that best fit the measured stress–strain curves. However, the strain energy density function is related to the internal structure of rubber. Kawabata proposed the following equation.

(10) $$W=C_{1}T\left(I_{1}-3\right)+\beta \left(I_{2}-3\right)+\emptyset {\left(I_{1}-3\right)}^{n}$$

**Figure 10:**
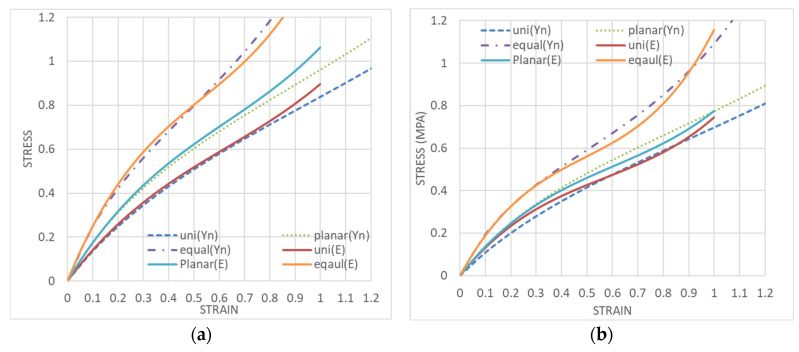
Approximate equation using the strain energy density function in Equation (8) proposed by Yamashita et al. compared with experimental values. (**a**) First elongation; (**b**) after 10 elongations.

Yamashita et al. proposed a more ingenious Formula (8), which is similar to the James–Green–Simpson Formula (7) [47]. Yamashita’s equation is devised to satisfy the two Equations (7) and (10). Here, C_10_ is assumed to be the term due to rubber elasticity, which is entropy elasticity; C_01_ is the term due to energy elasticity resulting from the entanglement of the polymer’s molecular chains; and C*_nm_*, *m*, and *n* are energy elastic terms resulting from the elongation of the polymer molecular chains by the addition of reinforcement. The coefficients of Yamashita’s equation were obtained from the stress–strain curves of the 1st and 11th elongations of silicone rubber that had each been de-entangled after 10 cycles of equal biaxial deformation. The results are shown in Figure 10 and Table 5.

The table clearly shows that the disentanglement effect contributes to both C_10_ and C_01_ in the Mooney–Rivlin equation. To obtain the best approximate solution, the values are obtained by numerical simulation without considering the meanings of the coefficients. Thus, how to incorporate such an algorithm into the simulation is a future issue.

Zheng Dongchang et al. [43] examined entanglement rupture, and if there are physical and entanglement crosslinking points, the C_10_ decrease may be caused by the rupture of molecular chains due to repeated elongation of the entanglement crosslinking points and the shedding of entanglement. C_01_ may be related to a decrease in the potential energy between molecular chains, which is lost when the entanglement bridging points settle in a sliding stable position due to repeated elongation. These possibilities would be difficult to elucidate without further verification by X-ray and neutron scattering.

### 4.2. Comparison of Apparent Strain between Chucks and Actual Strain between Rubber Marks in Biaxial Deformation

Several issues exist in biaxial deformation, such as the large number of chucks that grip the rubber, the initial sagging of the rubber sheet due to wrinkling caused by chucking, and the tendency of the rubber sheet to break at or between the chucks. However, this measurement is necessary to obtain the Ogden coefficient and the Mooney–Rivlin coefficient for CAE analysis. In addition, the results of uniaxial elongation dumbbell tests by the usual JIS and ASTM standards, and those of biaxial elongation tests, inevitably do not match due to differences in sample geometry. Therefore, it is important to compare the apparent displacement between chucks in the biaxial elongation test with the strain between points of the rubber itself.

**Figure 11:**
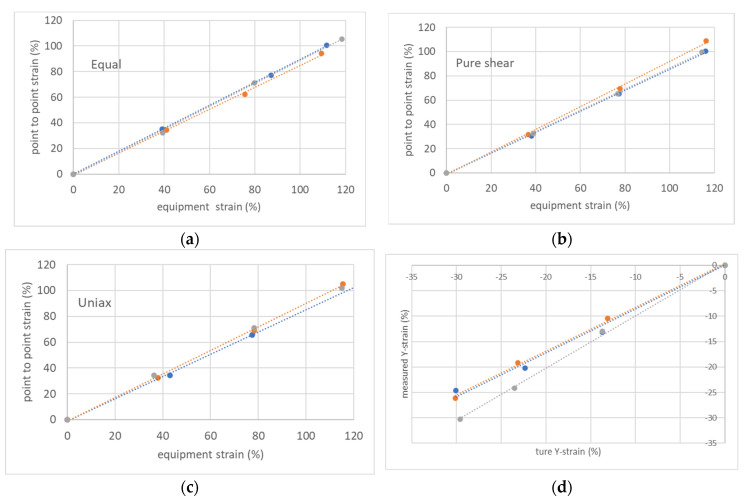
Relationship between the apparent strain of the rubber obtained from the device between chucks and the actual strain measured from markers drawn on the rubber. (**a**) Equal biaxial; (**b**) pure shear; (**c**) uniaxial; (**d**) comparison of measured values of shrinkage in the y-direction in uniaxial elongation concerning the theoretical true value when Poisson’s ratio of 0.5 is assumed.

Figure 11 shows that the strain between the points was 0.876 times the strain between the chucks in equal biaxial elongation, 0.878 times in uniaxial constrained biaxial, and 0.895 times in uniaxial. This may be due not only to slippage at the chuck in biaxial elongation but also to the larger deformation of the rubber at the chuck during the biaxial deformation of the rubber. In addition, the shrinkage of the shrinkage side not secured by the chuck in uniaxial elongation was on average 0.895 times larger than it should have been, indicating that the rubber did not shrink sufficiently for elongation at the chuck in broad uniaxial elongation (Figure 11d). This is also an issue for uniaxial elongation in biaxial tests and will require further study.

**Figure 12:**
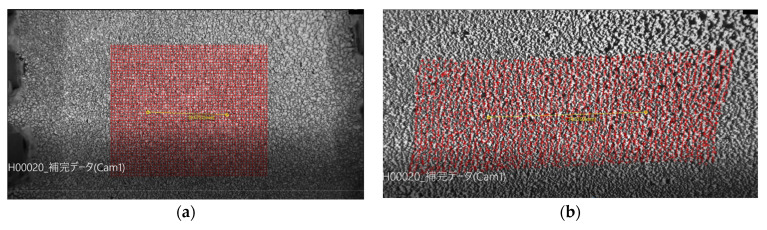

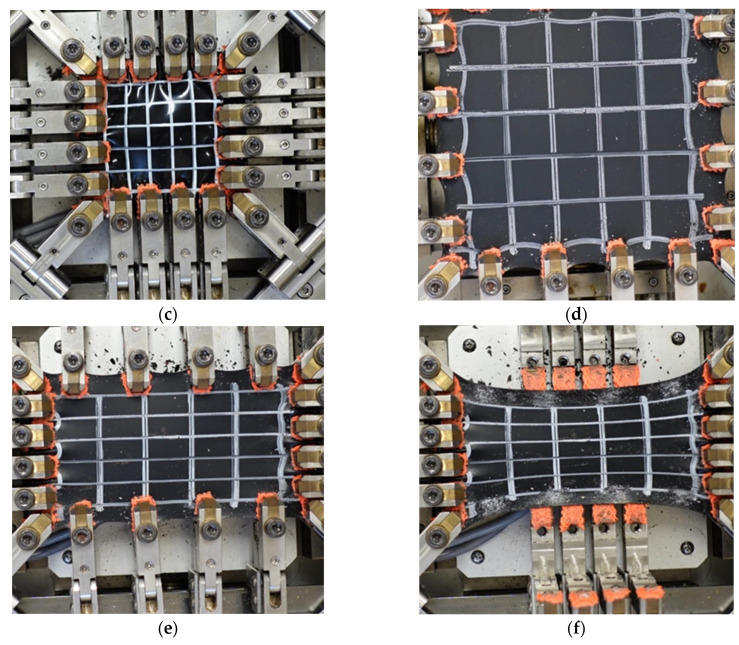
Comparison of digital image correlation (DIC) and direct image measurement methods for biaxial deformation strain. (**a**) DIC method undeformed; (**b**) DIC method uniaxial elongation; (**c**) undeformed condition in actual biaxial elongation; (**d**) equally axial deformation; (**e**) uniaxial constrained biaxial deformation; (**f**) uniaxial deformation.

The stereo digital image correlation method Stereo-DIC Dipp-Strain was also examined (Figure 12a,b). In uniaxial elongation, the strain between chucks was 118.5%, while the strain at the distance between points was 102.3% (Figure 12f), and it was 86.4% in stereo with the autodial image correlation method. The distance between chucks was 50 mm, the distance between points was 30 mm, and the measurement distance of the stereo digital image correlation method was 12 mm. The stereo image correlation method looks at the center of the sample, which may suggest that there may be less distortion in the center than in the entire sample, but there is also the issue of accuracy concerning the very shape. Since the silicone rubber used in this study has poor adhesive properties, the spray paint spots may crack and separate into two pieces due to elongation. For this reason, we did not use the strain from the stereo digital image correlation method but instead evaluated the distance between the spots as the strain of the sample.

**Figure 13:**
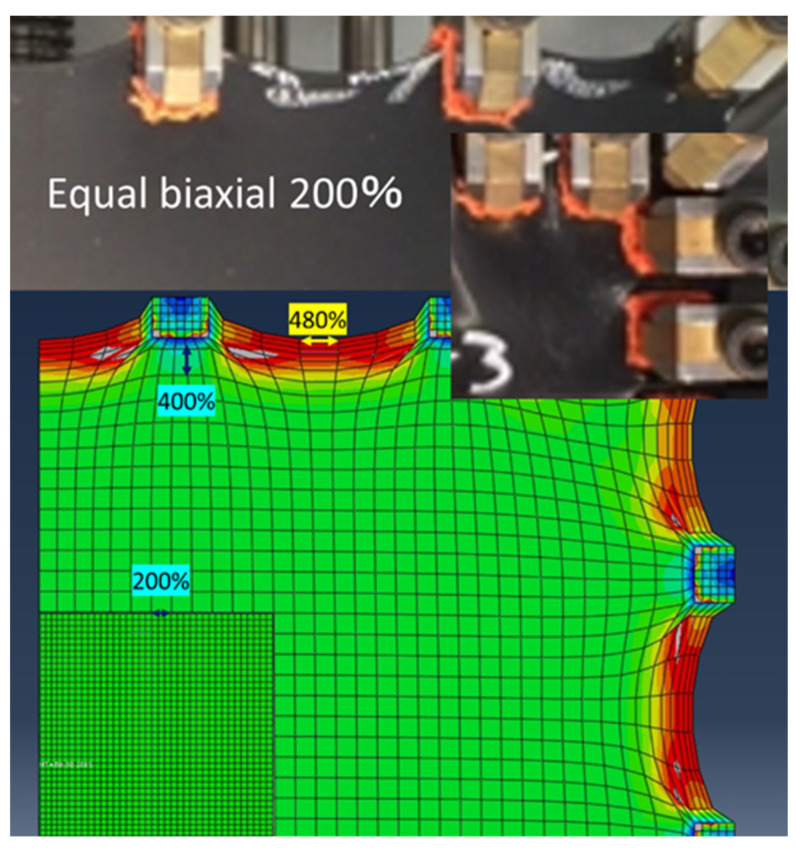
Strain near the chuck using Abaqus 6.14. Compared to the undeformed state (bottom left), deforming the rubber sheet by 200% in an equal biaxial manner results in a strain of 400% at the tip of the chuck and up to 480% between chucks.

In the biaxial measurements, the four chucks at the corners do not measure forces. From Figure 12d,e, it appears that almost uniform biaxial and uniaxially constrained biaxial deformation is applied to the front surface of the rubber sheet. As for uniaxial elongation, it is not completely uniaxial, as mentioned earlier. However, it is impossible to guess from the photograph. Therefore, hyperelasticity analysis was performed using CAE analysis. The parameter used was the Ogden coefficient after the 11th equal repetition of elongation in Table 5. As a result, it was estimated that, on average, 480% of the strain was applied to the area between the chucks, which is more than twice the strain at the center of the rubber. The strain at the tip of the chuck gripper was also 400% (Figure 13). Therefore, the measured values for the even biaxial were higher than the true stress at 200%. Fujikawa et al. pointed out that this apparent increase in the measured value is 35% to 40%. On the other hand, in the present analysis, the strain is higher than 200% only in a smaller range. However, this apparent increase in the measured value is thought to depend on the shape of the chuck, the amount of strain applied, and other factors. Therefore, from the analysis, we can estimate 65% for the 200% strain range, 20% for the 200–400% strain range, and 15% for the 400–480% strain range. In the future, when actual product behavior is compared between analysis and measurement using the strain energy density function identified from biaxial measurement if the analysis produces higher values than the measurement it will be necessary to consider whether such overestimation during measurement might be the cause.

### 4.3. Application to the Prediction of Stress Drop in Cyclic Fatigue of Practical Rubber Boots

**Figure 14:**
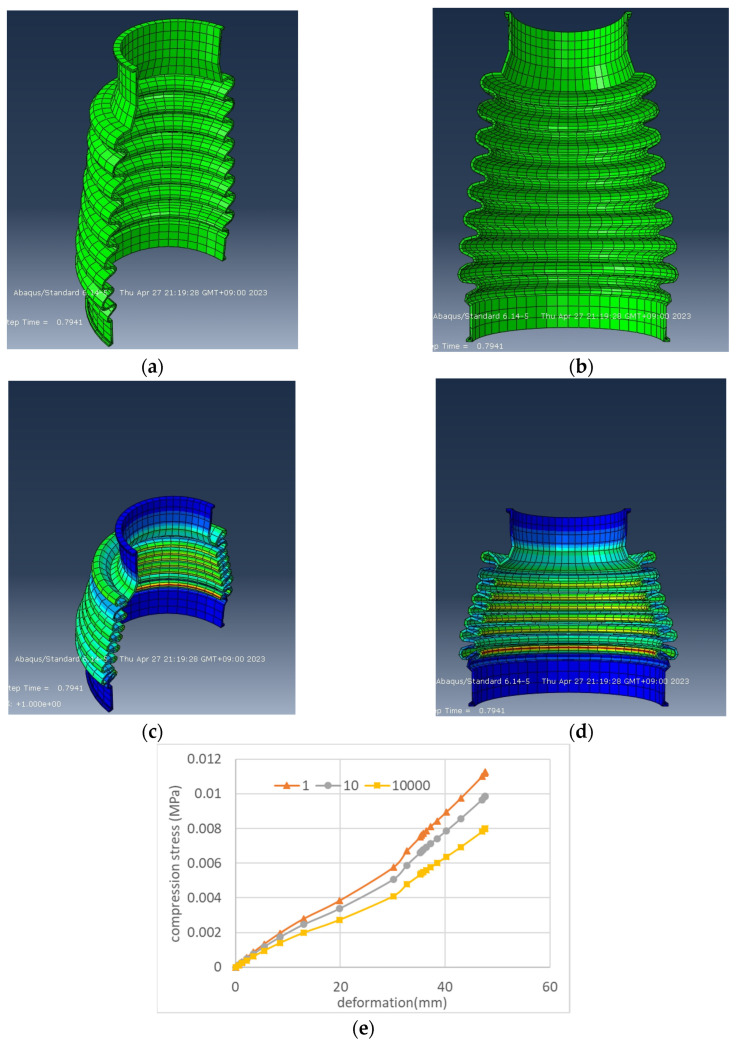
Abaqus prediction of compressive stress reduction due to cyclic deformation of rubber boots predicted using strain energy density functions obtained from biaxial deformation experiments: (**a**) cross-sectional perspective of the undeformed side; (**b**) cross-sectional perspective of the undeformed front; (**c**) side in compression after 10,000 cycles; (**d**) front in compression after 10,000 cycles; (**e**) reduction in stress after repeated compression of rubber boot.

In practical products made of rubber and elastomer materials, it is crucial to predict the fatigue characteristics resulting from repeated deformation. For example, the operating parts of a car are designed to move smoothly when force is transmitted and filled with grease (lubricant). The presence of a rubber boot prevents water, sand, and other debris from entering the moving parts and interfering with their movement. Using the numerical values obtained from the hyperelastic model (using the Mooney–Rivlin approximation in Table 2 and Table 3), we predicted the relationship between the amount of contraction deformation and compressive stress when the rubber boot is compressed 10,000 times, as shown in Figure 14e. It can be seen that the compressive stress of the rubber boot decreased by 2/3 with repeated deformation. Additionally, it shows that the stress is highest at the lowest part of the boot bellows and that there is a high possibility of a tear occurring in this area. If the stress concentration points of the rubber boot are known, the shape of the boot can be changed to make it more resistant to tearing, and silicone rubber of a different hardness can be used to increase durability, thus enabling product design without conducting experiments. The analysis of the rubber boot in Figure 14 is for compression, but the drive shaft boot is subjected not only to compression but also to bending deformation as the inside of the boot moves back and forth and side to side during vibration and steering wheel turning, so the load on the boot is greater than that on other boots. This technique can predict durability during long-term use.

## 5. Conclusions

This study elucidated the following.

1. The rubber material undergoes stress softening with each cyclic elongation, and the stress value settles down to a nearly constant value after about 10 cycles. Since actual rubber materials are constantly subjected to repeated deformation, it is necessary to calculate the strain energy density function under conditions where the stress is constant.
2. To calculate the correct strain energy density function of rubber materials, it is suitable to measure uniaxially constrained biaxial and uniaxially elongated stress–strain curves under conditions where the intramolecular entanglement and cohesive state of filled particles are dispersed throughout the rubber by repeated biaxial deformation.
3. From the decrease in the strain energy density function due to repeated elongation, the entanglement within the rubber molecular chains and the aggregation of the filled particles accounted for 25% of the total energy of the entire rubber.
4. The strain between adjacent chucks gripping the rubber sheet was found to be twice as great as that of the center parts of the rubber, but the measuring device has been devised not to detect the force applied between these chucks.

## Figures and Tables

**Table 1:** Comparison of the Ogden coefficient [23] and the Mooney–Rivlin coefficient [18] obtained by the measurement method shown in Figure 1.

	***μ*** **_1_**	***μ*** **_2_**	***μ*** **_3_**	***α*_1_**	***α*_2_**	***α*_3_**
Same sheet	5.97 × 10^−4^	7.75 × 10^−1^	−7.25 × 10^−2^	9.56 × 10^−0^	1.10 × 10^−0^	−1.84 × 10^−0^
Separate sheets	8.35 × 10^−3^	1.19 × 10^−0^	−5.74 × 10^−4^	6.17 × 10^−0^	8.35 × 10^−1^	−5.04 × 10^−0^
		C_10_	C_01_	C_11_	C_20_	C_30_
Same sheet		1.71 × 10^−1^	7.69 × 10^−2^	−1.47 × 10^−2^	2.98 × 10^−3^	4.51 × 10^−3^
Separate sheets		2.16 × 10^−1^	5.19 × 10^−2^	−1.12 × 10^−2^	−1.28 × 10^−3^	4.42 × 10^−3^

**Table 2:** Comparison of Ogden coefficient [23] and Mooney–Rivlin coefficient [18] after repeated elongation to loosen entanglement and silica aggregation within rubber molecules.

**Repeat**	***μ*** **_1_**	***μ*** **_2_**	***μ*** **_3_**	***α*_1_**	***α*_2_**	***α*_3_**
1	1.57 × 10^−0^	6.27 × 10^−2^	−2.95 × 10^−4^	5.08 × 10^−1^	3.79 × 10^−0^	−5.48 × 10^−0^
2	6.68 × 10^−1^	5.49 × 10^−2^	−5.82 × 10^−5^	9.89 × 10^−1^	3.98 × 10^−0^	−7.37 × 10^−0^
3	7.53 × 10^−3^	6.88 × 10^−1^	−3.88 × 10^−5^	6.49 × 10^−0^	1.17 × 10^−0^	−7.52 × 10^−0^
5	6.04 × 10^−1^	7.89 × 10^−5^	−6.54 × 10^−5^	1.46 × 10^−0^	1.34 × 10^+1^	−6.84 × 10^−0^
10	−2.81 × 10^−2^	4.22 × 10^−1^	−1.50 × 10^−4^	1.78 × 10^−0^	2.12 × 10^−0^	−6.51 × 10^−0^
Repeat		C_10_	C_01_	C_11_	C_20_	C_30_
1		2.17 × 10^−1^	4.27 × 10^−2^	−7.53 × 10^−3^	1.34 × 10^−3^	2.87 × 10^−3^
2		1.91 × 10^−1^	5.24 × 10^−2^	−2.40 × 10^−2^	−2.44 × 10^−3^	1.13 × 10^−2^
3		1.88 × 10^−1^	4.85 × 10^−2^	−2.30 × 10^−2^	−2.88 × 10^−3^	1.09 × 10^−2^
5		1.89 × 10^−1^	4.66 × 10^−2^	−2.32 × 10^−2^	−2.22 × 10^−3^	1.07 × 10^−2^
10		1.86 × 10^−1^	4.25 × 10^−2^	−2.16 × 10^−2^	−2.06 × 10^−3^	1.00 × 10^−2^

**Table 3:** Predicted Mooney–Rivlin coefficients after repeated elongation.

Repetitions	C_10_	C_01_	C_11_	C_20_	C_30_
100	1.82 × 10^−1^	2.83 × 10^−2^	−1.88 × 10^−2^	−1.06 × 10^−3^	8.12 × 10^−3^
10,000	1.73 × 10^−1^	1.20 × 10^−2^	−1.28 × 10^−2^	−1.00 × 10^−4^	6.00 × 10^−3^

**Table 4:** Ogden coefficient [23] and Mooney–Rivlin coefficient [18] obtained from the 11th measurement data in which repeated elongation is performed and the relaxation behavior of the rubber is considered to be in a pseudo-equilibrium state.

**Repeat**	***μ*** **_1_**	***μ*** **_2_**	***μ*** **_3_**	***α*_1_**	***α*_2_**	***α*_3_**
11 (10 times equal repeat)	3.55 × 10^−1^	5.00 × 10^−4^	−4.13 × 10^−3^	2.11 × 10^−0^	2.58 × 10^−0^	−3.67 × 10^−0^
11 (10 times indivisible repeat)	3.42 × 10^−1^	8.54 × 10^−2^	−1.35 × 10^−4^	1.40 × 10^−0^	3.38 × 10^−0^	−5.98 × 10^−0^
Repeat		C_10_	C_01_	C_11_	C_20_	C_30_
11 (10 times equal repeat)		2.03 × 10^−1^	6.99 × 10^−3^	−2.10 × 10^−3^	−8.68 × 10^−3^	2.89 × 10^−3^
11 (10 times indivisible repeat)		1.97 × 10^−1^	1.48 × 10^−2^	−8.71 × 10^−3^	−3.48 × 10^−3^	4.78 × 10^−3^

**Table 5:** Coefficients of the strain energy density function in Equation (8) proposed by Yamashita et al.

	C_10_	C_01_	C*_nm_*	*n*	*m*
1	0.22	0.03	0.001	2	−0.2
11 (equal)	0.19	0.01	0.001	2	−0.2

## Data Availability

The raw data of the figures of this study can be provided in an Excel file. If you wish to receive these data, please contact the first author, Yoshihiro Yamashita, by e-mail. The data will also be made available for download from the website of the Center for Fibers and Materials Research at the University of Fukui after the paper is published at http://yamashita-777.sakura.ne.jp/saporting_page.html (accessed on 8 May 2023).
